# Usefulness of Glucose Monitoring in Neonates of Mothers With an Elevated Glucose Challenge Test and a Normal Oral Glucose Tolerance Test

**DOI:** 10.7759/cureus.77244

**Published:** 2025-01-10

**Authors:** Margaret A Uchefuna, Giddel Alvarado, Imoh L Ebong, Saman Aryal, Sheng-Hsin Chen, Alexander Rodriguez

**Affiliations:** 1 Department of Pediatrics, NYC Health+Hospitals/Woodhull Medical Center, New York, USA

**Keywords:** blood glucose monitoring, elevated glucose challenge test, gestational diabetes, hypoglycemia, low blood glucose, metabolic, neonatal hypoglycemia, newborns, normal oral glucose tolerance test

## Abstract

Background

Neonatal hypoglycemia is a common metabolic disturbance in neonates. Glucose monitoring is recommended for certain groups of neonates, including those whose mothers have pre-gestational or gestational diabetes. Little is known about the relevance of glucose monitoring in neonates whose mothers have an elevated screening glucose challenge test (GCT) but a normal oral glucose tolerance test (OGTT).

Objectives

The objectives of this study were to determine if neonates of mothers with an elevated GCT but a normal OGTT had hypoglycemia and to establish if there was an association between the maternal GCT and neonatal blood glucose level.

Methods

A single-site retrospective analysis was conducted on 307 neonates born in a community hospital in the Brooklyn area of New York between November 1, 2021, and November 1, 2023, who were identified as being at risk for hypoglycemia due to known risk factors like maternal diabetes, prematurity, low birth weight, and small or large size for gestational age, as well as possible risk factors like an elevated maternal GCT but a normal OGTT. Glucose monitoring had been done for these neonates at birth per AAP recommendations. The Office of Science and Research Institutional Review Board reviewed the study which was determined to meet the criteria for exemption. The individual authorization requirements were waived and adequate steps were taken to ensure data privacy. Neonates of diabetic mothers or mothers with an elevated OGTT and neonates who were born preterm, low birth weight, and small or large for gestational age were excluded from the study while neonates whose mothers had an elevated GCT, but a normal OGTT were included. This brought to 63 the number of neonates who met the inclusion criteria. Charts were reviewed to see if these neonates had hypoglycemia during the course of glucose monitoring. The chi-square test was used for categorical variables and the two-sample *t-*test was used for continuous variables.

Results

In our study, eight of the 63 neonates had asymptomatic hypoglycemia while the rest were euglycemic. None required admission to the neonatal intensive care unit and the hypoglycemia resolved with oral feed, oral glucose gel, or a combination of the two. Although hemoglobin A1c was also elevated in eight of the 63 mothers, all the mothers of the neonates with hypoglycemia had normal hemoglobin A1c, and no relationship was found between the hypoglycemic reaction (X^2^ = 0.927) and hemoglobin A1c in the mothers who had an elevated GCT but a normal OGTT. The male-to-female ratio was approximately 1:1, and there was no relationship between gender and neonatal hypoglycemic reaction (X^2^ = 0.002). There was also no correlation between maternal GCT and initial (r=-0.173) or lowest (r=-0.182) neonatal glucose readings.

Conclusion

Our study demonstrated that the likelihood of hypoglycemia in neonates of mothers with an abnormal GCT, but a normal OGTT was slim. Further studies are needed, and a larger group size would be of benefit.

## Introduction

Hypoglycemia is one of the most frequent metabolic imbalances that occur in neonates [1] and is a common reason for neonatal hospitalizations worldwide [2]. It affects approximately 5-15% of otherwise healthy neonates [3].

In utero, the fetus does not generate glucose within its body but relies entirely on maternal glucose obtained through the placental connection. The process of building glycogen stores through glycogenesis occurs slowly in the fetus in the early weeks of pregnancy but gradually increases towards term. At birth, the clamping of the cord disrupts glucose supply to the neonate, and hormone levels rise in an attempt to mobilize glucose through glycogenolysis and gluconeogenesis. This process may take a while which explains why healthy neonates usually experience a period of transitional hypoglycemia during the first 2-3 hours after birth [4]. The inability to normalize blood glucose levels over time poses risks for neurological complications like mental retardation, epilepsy, cardiac issues, and muscle weakness [5].

The American Academy of Pediatrics (AAP) defines neonatal hypoglycemia as blood glucose levels below specific thresholds, 40 mg/dL within the first four hours of life and 45 mg/dL between 4 and 24 hours of life and recommends screening glucose checks for certain groups of neonates who, based on different mechanisms, are at risk for hypoglycemia [6]. This includes neonates who are born late preterm, small or large for gestational age, or whose mothers have pre-gestational or gestational diabetic mothers.

Gestational diabetes mellitus (GDM) is the onset of diabetes during pregnancy, in women who did not have diabetes pre-pregnancy. In a recent meta-analysis, the global prevalence was found to be 4.4%, higher in the US and Canada compared to Europe [7]. According to the American Diabetes Association, the evaluation of gestational diabetes involves a two-step approach. Initially, a non-fasting glucose challenge test (GCT) is performed between 24 and 28 weeks of pregnancy. An elevated test result defined by a one-hour random glucose equal to or greater than 135 mg/dl after ingesting a 50-g glucose load is followed by a confirmatory fasting oral glucose tolerance test (OGTT) which entails drinking a 100-g glucose load. Gestational diabetes is diagnosed when two or more values from the OGTT are elevated above certain cut-offs based on widely accepted criteria [8]. For our study, we used the Carpenter-Coustan criteria, in keeping with our hospital's guidelines, to identify an abnormal OGTT, with values defined as elevated if they were equal to or greater than 95 mg/dL for fasting glucose, 180 mg/dL for one-hour glucose, 155 mg/dL for two-hour glucose and 140 mg/dL for three-hour glucose [9].

In some mothers, the elevated screening GCT is followed by a normal OGTT, creating a gray area in some centers as to whether glucose monitoring is required in the neonate, or not. Our study aimed to assess the usefulness of glucose monitoring in asymptomatic neonates born to mothers with elevated GCTs but normal OGTT results.

## Materials and methods

A single-site retrospective analysis was conducted on 307 neonates born in a community hospital (Woodhull Medical Center) in the Brooklyn area of New York between November 1, 2021, and November 1, 2023, who were identified as being at risk for hypoglycemia due to known risk factors like maternal diabetes, prematurity, low birth weight, small or large size for gestational age, as well as possible risk factors like an elevated maternal GCT but a normal OGTT. Glucose monitoring had been done for these neonates at birth per AAP recommendations. The Office of Science and Research Institutional Review Board reviewed the study which was determined to meet the criteria for exemption. The individual authorization requirements were waived and adequate steps were taken to ensure data privacy.

**Figure 1:**
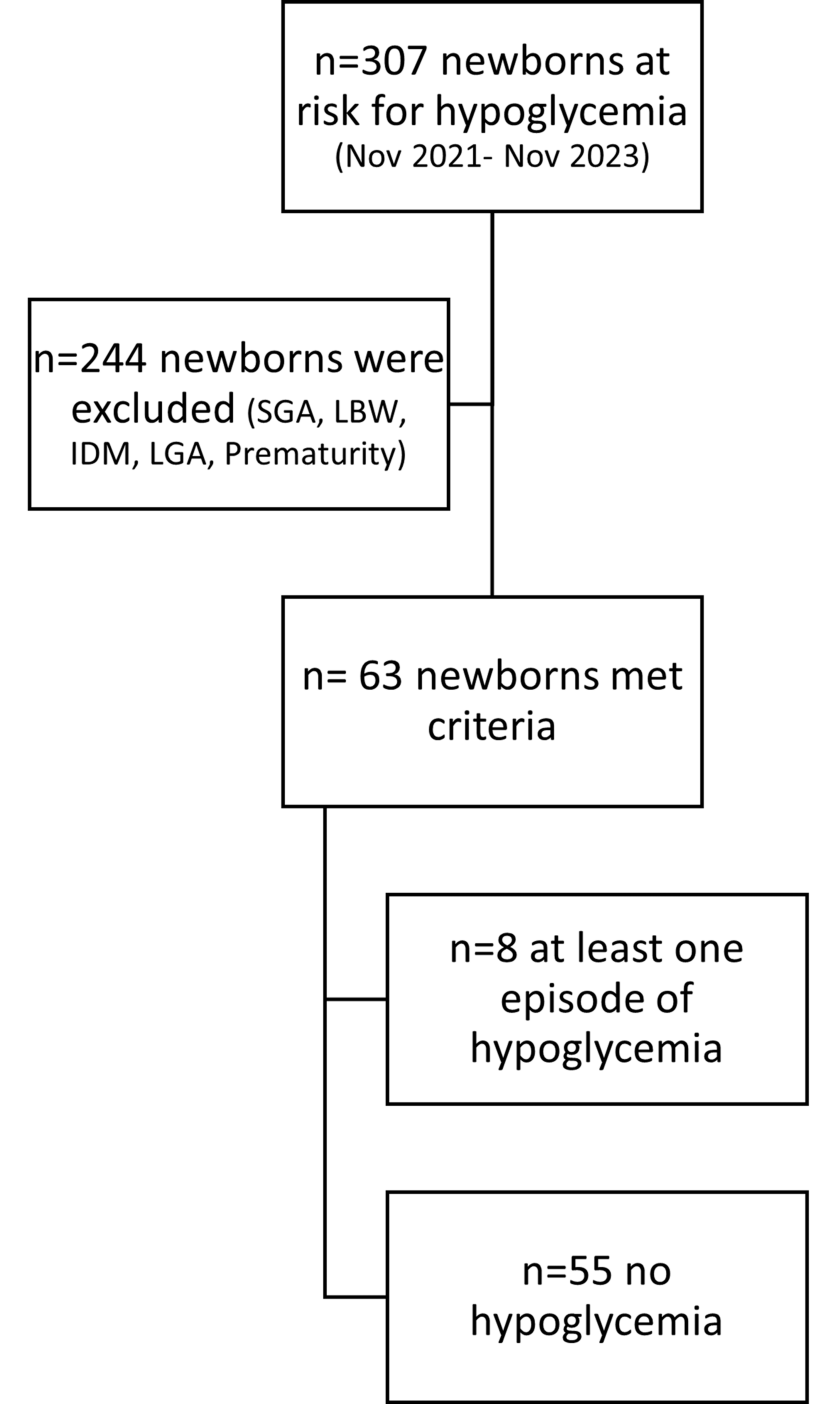
Chart review methodology SGA: small for gestational age, LBW: low birth weight, IDM: infants of diabetic mothers, LGA: large for gestational age

Neonates of diabetic mothers or mothers with elevated OGTT and neonates who were born preterm, low birth weight, small or large for gestational age were excluded from the study while neonates whose mothers had an elevated GCT but a normal OGTT were included. This brought to 63 the number of neonates who met the inclusion criteria (Figure 1).

An elevated non-fasting 50-g, one-hour GCT was plasma glucose ≥ 135 mg/dl (defined by the American College of Obstetricians and Gynecologists criteria for areas with a high prevalence of GDM). The charts of neonates who met the inclusion criteria were reviewed to see if they had hypoglycemia at the time of glucose monitoring during hospital stay. Neonatal hypoglycemia was defined as capillary blood glucose level <40 mg/dl between 0 and 4 hours of life and < 45 mg/dl between 4 and 24 hours of life [6]. 

For statistical processing, the chi-square test was used for categorical variables and the two-sample t-test was used for continuous variables. Multiple logistic regression analysis was performed to estimate the odds ratio (ORs) of adverse outcomes with adjustment for confounders. Statistical analysis was performed using Statistical Analysis System (SAS) version 9.2 (SAS Institute, Inc., Cary, NC). 

## Results

A sample of 63 women and their neonates were included in the final analysis. The women were of diverse ethnicity although 41 (65.1%) identified primarily as Hispanic or Latinx. The average age of the women was 30.65 years (SD = 6.34 years). All the GCT results were elevated with an average of 156.78 mg/dl (SD = 22.08), while the OGTT results were in the normal range, per study protocol. More than three-quarters of the mothers’ hemoglobin A1c readings were in the normal range while 8 mothers (12.7%) had elevated readings. The male-to-female ratio was approximately 1:1, the average birthweight was 3,212.40 grams (SD = 375.65 grams) and the average gestational age was 39.20 weeks (SD = 1.17 weeks). Fifty-five (87.3%) of the 63 neonates had no hypoglycemia, while 8 (12.7%) had hypoglycemia (see Tables 1, 2).

**Table 1:** Sample demographics

Variable	Frequency	Percentage	
Gender			
Female	32	50.8%	
Male	31	49.2%	
Total	63	100.0%	
Race/Ethnicity			
Black/African American	7	11.1%	
Hispanic/Latinx	41	65.1%	
Jewish	1	1.6%	
Not Hispanic	3	4.8%	
Asian	1	1.6%	
Russian	4	6.3%	
White	1	1.6%	
Unspecified/Unknown/Missing	5	7.9%	
Total	63	100.0%	
Hypoglycemia			
Negative	55	87.3%	
Positive	8	12.7%	
Total	63	100.0%	
Maternal GTT			
Normal	63	100%	
Missing	0	0%	
Total	63	100%	
Maternal HbA1c			
Normal	49	77.8%	
Elevated	8	12.7%	
Missing	6	9.5%	
Total	63	100.0%	

**Table 2:** Sample statistics GCT: glucose challenge test

Variable	Mean	Standard Deviation
Birthweight (in grams)	3,212.40	375.65
Gestational age (in weeks)	39.20	1.17
Maternal age (in years)	30.65	6.34
Maternal GCT	156.78	22.08

Of the eight babies with hypoglycemia, two babies (25%) had one hypoglycemic value each, four babies (50%) had two hypoglycemic values each, and two babies (25%) had three hypoglycemic values each. Thus, 16 hypoglycemic readings were distributed among the eight babies, with two (12.5%) occurring before four hours of life and 14 (87.5%) between 4 and 24 hours of life. The average initial glucose reading was 64.83 mg/dl (SD=18.74), and the average lowest glucose reading of the babies was 53.50 mg/dl (SD=10.57). All the neonates with hypoglycemia were identified within the first three glucose measurements, per the AAP hypoglycemia screening protocol.

In the next set of analyses, we tested to determine if there were differences in birthweight, maternal GCT, and gestational age as a function of hypoglycemia in the neonate. Independent t-test analyses demonstrated no relationship between neonatal hypoglycemia and birthweight, maternal GCT, or gestational age (see Table 3).

**Table 3:** Differences in birthweight, maternal GCT, and gestational age as a function of hypoglycemia in neonates GCT: glucose challenge test; NS: not significant

Test variable	Hypoglycemic Reaction	Mean	SD	T	P
Birthweight	Negative	3,250.80	388.79		
	Positive	3,184.63	281.73	0.463	NS
Maternal GCT	Negative	156.11	21.23		
	Positive	161.38	28.51	-0.627	NS
Gestational age	Negative	39.13	1.16		
	Positive	39.70	1.21	-1.278	NS

Our study also found no correlation between elevated maternal GCT (in the setting of normal OGTT) and neonatal hypoglycemia (see Table 4).

**Table 4:** Correlations between GCT levels and glucose screenings GCT: glucose challenge test; NS: not significant

	Pearson Correlation	P
Correlation between maternal GCT and Initial glucose reading	-0.173	NS
Correlation between maternal GCT and lowest glucose reading	-0.182	NS
Correlation between initial and lowest glucose readings	0.719	<0.001

## Discussion

Most babies with hypoglycemia are asymptomatic which is where the real concern lies as subtle neurological damage may occur before symptoms become evident [10]. The symptoms of hypoglycemia are non-specific and include apnea, jitteriness, irritability, poor feeding, poor tone, hypothermia, and seizures. Treatment interventions for hypoglycemia are recommended parenterally for all symptomatic neonates and when oral supplementation fails for asymptomatic neonates whose blood glucose levels fall below the defined cut-offs per hour of life, as stated by current guidelines [6].

A 2024 publication by Andrews et al. concluded that neonates whose mothers had an abnormal glucose screen test (even if the confirmatory OGTT was unremarkable) were more likely to have hypoglycemia when compared to those whose mothers had a normal glucose screen [11]. This was similar to other studies [12,13] in similar settings which showed a more frequent occurrence of hypoglycemia. Our study found an average hypoglycemic value of 37.5 mg/dl within four hours of life and 40 mg/dl between 4 and 24 hours of life, and none of the neonates was symptomatic.

Women with elevated glucose screening tests were also found more likely to have newborns who were larger in size for gestational age and required cesarean section or assisted delivery and this occurred despite subsequent normalization of maternal glucose values [14-17]. The babies in our study had no significant difference in birth weight as a function of hypoglycemia.

Early pregnancy causes a hormonal surge which leads to insulin resistance; some studies have shown the insulin-resistant states of gestational hyperglycemia to be associated with worse neonatal outcomes and greater need for neonatal ICU admissions when compared to insulin-deficient states [16]. None of the hypoglycemic neonates in our study required IV fluids or neonatal ICU admission as there was resolution of hypoglycemia with oral feed, oral glucose gel, or a combination of both.

Other negative perinatal outcomes like hyperbilirubinemia, birth trauma, and stillbirth have also been found to occur in neonates of women with impaired glucose tolerance of any form [12,13]. This finding is relatable to several other studies that have shown an increased risk for untoward outcomes in neonates of women with impaired glucose tolerance of any form [18-20]. In contrast, a remarkably decreased incidence of low blood glucose was seen in a study by Wang et al. [21] of neonates whose mothers had impaired glucose tolerance when compared to those whose mothers had gestational diabetes mellitus.

Our study showed a 1:1 ratio in gender distribution among the neonates with hypoglycemia and no significant relationship was found between gender and hypoglycemic reaction (X2 = 0.002). Although no correlation was seen between elevated maternal GCT (in the setting of normal OGTT) and neonatal hypoglycemia, the mean difference between the initial and lowest glucose values was found to be statistically significant (t=6.812, p<0.001). This great variability in readings following the initial test shows that although the first glucose check may be normal, subsequent checks can still be low which signals the need for serial glucose checks in those neonates who require glucose monitoring until they are able to fully establish their own glucose homeostasis. There is yet no consensus on whether elevated maternal GCT with subsequent normal maternal OGTT is a risk factor for neonatal hypoglycemia, or whether glucose monitoring is beneficial in neonates of such mothers. Our study helped provide additional research data that supports the idea that there may be a need to monitor glucose levels in this group.

In addition, we found that eight (12.7%) out of the 63 mothers had an elevated hemoglobin A1c (hbA1c). Interestingly, all the mothers of the babies with hypoglycemia had normal hbA1c, and we saw no relationship between the hypoglycemic reaction (X2 = 0.927) and hemoglobin A1c in the mothers who had elevated GCT but normal OGTT. Our study demonstrated no significant relationship between elevated maternal GCT (if OGTT is normal) and neonatal hypoglycemia and a relationship of great significance between the mean initial and lowest glucose values. However, our study was limited by its small sample size and the lack of a comparison group.

## Conclusions

Hypoglycemia is a common metabolic problem in neonates and can manifest very subtly. With changes in diet and an increase in the prevalence of mothers with elevated GCTs with or without gestational diabetes, more neonates may be at risk.

Our study demonstrated that the likelihood of hypoglycemia in neonates of mothers with an abnormal GCT but a normal glucose tolerance test was slim. Further studies are needed, and a larger group size would be of benefit.
